# The Absorption, Storage, and Transport of Ocular Carotenoids and Retinoids

**DOI:** 10.1146/annurev-vision-102122-101846

**Published:** 2024-09-02

**Authors:** Johannes von Lintig, Sepalika Bandara

**Affiliations:** Department of Pharmacology, School of Medicine, Case Western Reserve University, Cleveland, Ohio, USA;

**Keywords:** vision, carotenoids, retinoids, transport, binding proteins, receptors

## Abstract

Carotenoids, yellow and red pigments found abundantly in nature, play essential roles in various aspects of human physiology. They serve as critical molecules in vision by functioning as antioxidants and as filters for blue light within the retina. Furthermore, carotenoids are the natural precursors of vitamin A, which is indispensable for the synthesis of retinaldehyde, the visual chromophore, and retinoic acid, a small molecule that regulates gene expression. Insufficient levels of carotenoids and retinoids have been linked to age-related macular degeneration and xerophthalmia, respectively. Nevertheless, the mechanisms by which the eye maintains carotenoid and retinoid homeostasis have remained a mystery. Recent breakthroughs identified the molecular players involved in this process and provided valuable biochemical insights into their functioning. Mutations in the corresponding genes disrupt the homeostasis of carotenoids and retinoids, leading to visual system pathologies. This review aims to consolidate our current understanding of these pathways, including their regulatory principles.

## 1. INTRODUCTION

Carotenoids are pigments ranging from yellow to red that are synthesized by plants, fungi, and bacteria. These compounds, like other isoprenoids, are formed through the condensation of isoprene (C5) units (Moise et al. 2014). The C40 carbon skeleton of phytoene is the basic chemical structure of most carotenoids. The introduction of double bonds into this carbon skeleton leads to the formation of the extended polyene chromophore and the red color of lycopene. When the ends of the carbon skeleton of lycopene are cyclized, they create the β- and ε-ionone rings found in α-carotene (β,ε) and β-carotene (β,β), respectively. Further chemical modifications, such as hydroxylation of the rings and shifts of double bonds, give rise to new molecules, contributing to the vast diversity within this compound class. Apocarotenoids are produced through the oxidative cleavage of specific double bonds in the polyene chain of the parent carotenoid structure and include the substance class of retinoids. Retinoids comprise all natural and synthetic compounds with structural resemblance to all-*trans*-retinol, with or without the biological activity of vitamin A (Sporn et al. 1976). Collectively, the chemical alterations to the basic structure of carotenoids result in the plethora of carotenoids found in nature, with over a thousand distinct compounds identified.

One of the best-known carotenoids is β-carotene. Many other carotenoids also bear common names like lycopene, lutein, zeaxanthin, and astaxanthin. These names often predated the determination of their precise chemical structures. To streamline nomenclature, the International Union of Pure and Applied Chemistry has issued a comprehensive system for naming carotenoids, with hundreds having been identified and more discoveries on the horizon. An online database, searchable based on various factors, including chemical structures, is actively maintained and updated to catalog these compounds (Yabuzaki 2017).

**Figure 1:**
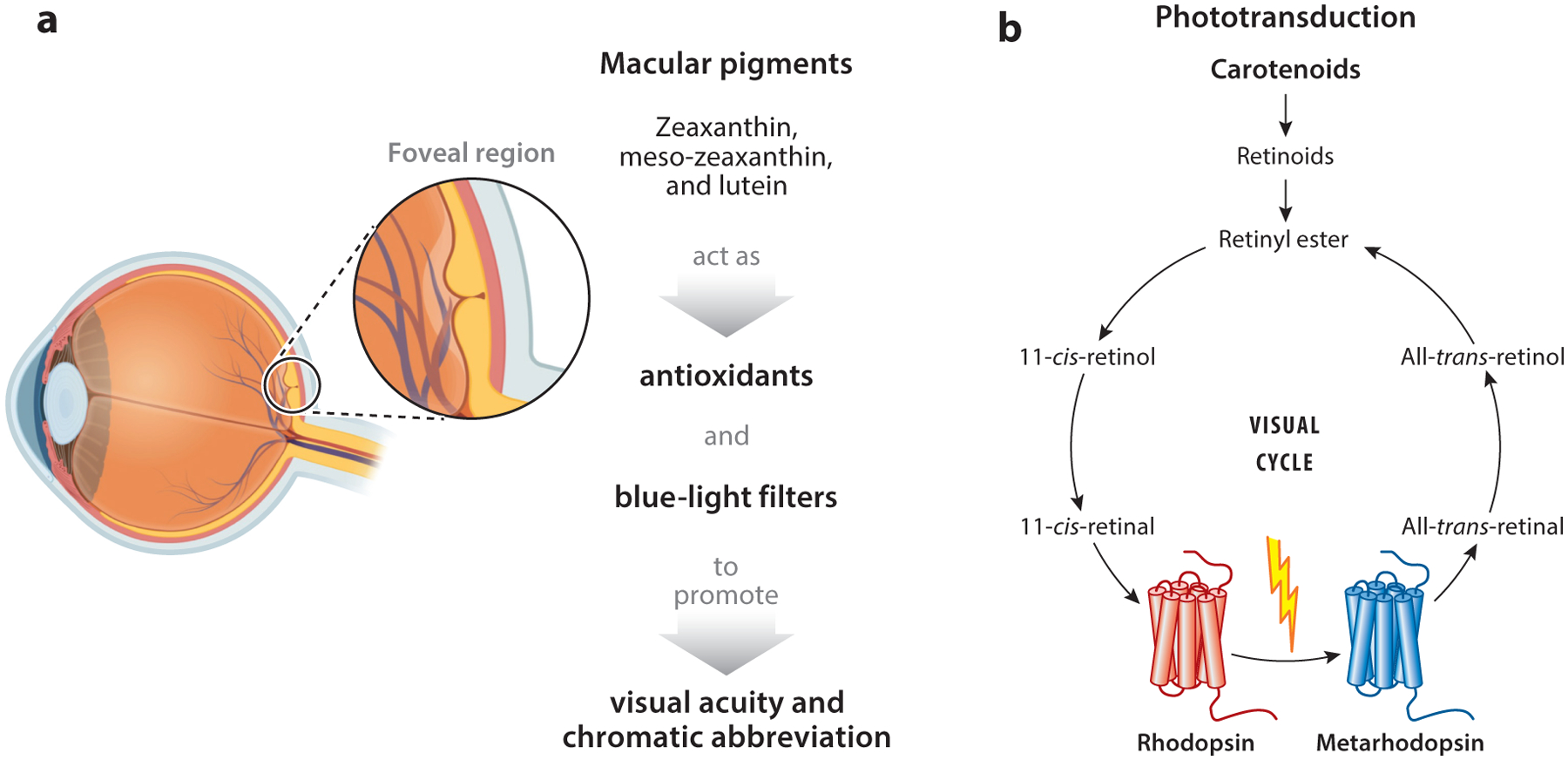
Ocular functions of macular pigments and retinoids. (*a*) The macular pigments zeaxanthin (3R,3′R-dihydroxy-β,β-carotene), meso-zeaxanthin (3R,3′S-dihydroxy-β,β-carotene), and lutein (3R,3′R-dihydroxy-β,ε-carotene) are amassed in the fovea centralis, where they serve as blue-light filters and antioxidants in cone photoreceptors and other retinal cells. (*b*) Retinoids stem from carotenoid precursors and are converted to chromophore (11-*cis-*retinal) in the eyes. 11-*cis-*Retinal binds to the opsin moiety to form functional visual pigments. Visual pigments are G protein–coupled receptors that mediate phototransduction. After bleach, 11-*cis-*retinal is converted to all-*trans*-retinal, which is recycled back to 11-*cis-*retinal through an enzymatic pathway called the visual cycle. Figure adapted from images created using BioRender.com.

Ongoing research examines the pivotal role of carotenoids and retinoids in vision (Bernstein et al. 2016, Kiser & Palczewski 2016, Mares 2016) (Figure 1). Insufficient carotenoid and vitamin A levels have been linked to conditions like xerophthalmia and degenerative eye diseases, including age-related macular degeneration (AMD) (Mares 2016, Sommer 2008). Several mechanisms have been proposed to elucidate how carotenoids can support eye health in humans. One key mechanism involves carotenoids’ capacity to act as antioxidants, particularly as free radical scavengers, within lipid-rich environments such as cell membranes and lipoproteins. This antioxidant action can help diminish lipid peroxidation, ultimately reducing oxidative stress and inflammatory responses in the cells of the neural retina and retinal pigment epithelium (RPE) (Bernstein et al. 2016, Mares 2016).

Carotenoids also provide a protective shield for human eyes by filtering out blue light, mitigating environmental stress on cellular components (Bernstein et al. 2016, Mares 2016). The macular pigments responsible for this function—lutein, zeaxanthin, and meso-zeaxanthin—have been identified chemically and are abundant in the primate retinal fovea, giving it a distinct yellow hue (Snodderly et al. 1991). Consequently, the fovea is often referred to as the macula lutea or the yellow spot. These pigments absorb short-wavelength blue light, potentially safeguarding photoreceptors from harm (Snodderly 1995, Widjaja-Adhi et al. 2018). Additionally, carotenoids help reduce the adverse effects of light scattering and chromatic aberration, thereby optimizing the retina’s contrast sensitivity (Hammond et al. 2013). Notably, nutritional supplementation and diets rich in lutein and zeaxanthin readily affect the concentrations of macular pigments in human eyes and reduce the risk of progression of the dry form of AMD to advanced stages in clinical studies (Chew et al. 2014).

Crucially, carotenoids and their retinoid metabolites are indispensable nutrients throughout one’s life. Investigations of the molecular underpinnings of these nutrients’ actions have led to the discovery of visual pigments and nuclear hormone receptors (Chambon 1996, Wald 1968). In this context, dietary provitamins like β-carotene, α-carotene, and β-cryptoxanthin are converted into retinaldehyde, a critical step in vitamin A synthesis. The 11-*cis-*retinal diastereomer binds to the G protein–coupled receptor component of cone and rod visual pigments via a Schiff’s base linkage. These complexes mediate phototransduction in the cone and rod photoreceptors of the retina (Kiser et al. 2014). Retinaldehyde is also transformed into retinol (vitamin A), retinyl esters (REs), and retinoic acid (RA) by endogenous enzymes in the eyes and other tissues throughout the body (von Lintig 2010). Retinol and REs serve as transport and storage forms of vitamin A (D’Ambrosio et al. 2011). They are stored in high concentrations in hepatic stellate cells but also in white adipose tissue and the lungs. Lipoproteins in the bloodstream transport REs, levels of which surge after a meal rich in precursor molecules. Retinol bound to a carrier protein predominates in the postmeal circulation, maintaining a homeostatic balance in the blood. RA is present in nanomolar concentrations in the blood, and its enzymatic synthesis is meticulously regulated through feedback mechanisms. RA binds to the RA receptor, which forms a dimer with the retinoid X receptor (Kedishvili 2013). These nuclear receptors are ligand-activated transcription factors governing the expression of genes associated with processes such as cell differentiation, embryonic eye development, immunity, and metabolism (Blaner 2019, Hall et al. 2011, Rhinn & Dollé 2012). This multifaceted action underscores carotenoids’ role as dietary precursors for hormones in the body and visual chromophores in photoreceptors of the eyes.

**Figure 2:**
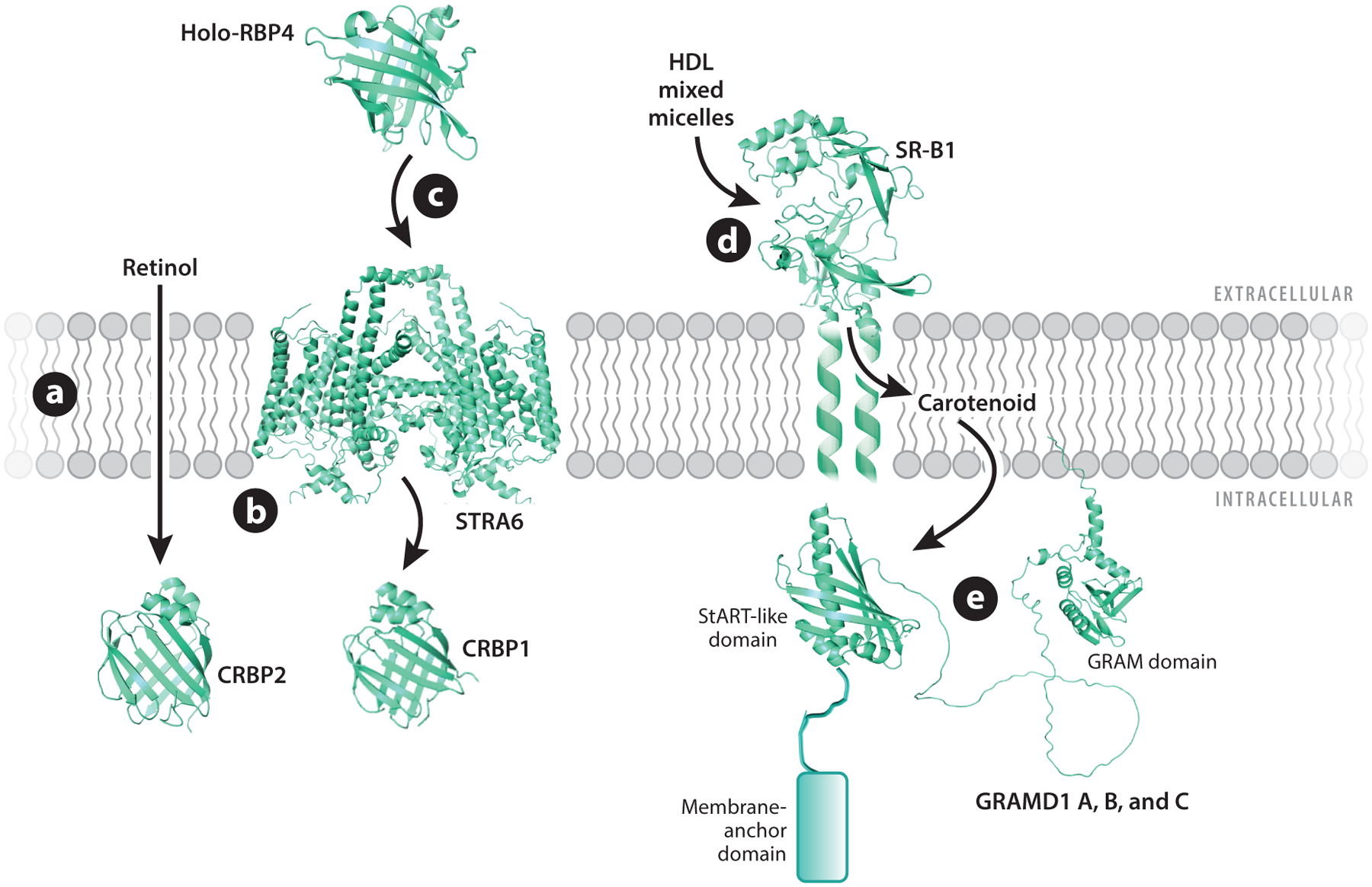
Transport modes of carotenoids and retinoids in mammals. (*a*) Retinol can cross membranes via passive diffusion. (*b*) Intracellular transport of retinol is facilitated by the retinol-binding proteins (RBPs) 1 (in hepatocytes, epithelial cells, and others) and 2 (in intestinal enterocytes). (*c*) Retinol is transported in the blood bound to RBP4, also known RBP. Holo-RBP4 binds with high affinity to a dimeric receptor encoded by the stimulated by retinoic acid 6 (*STRA6*) gene. The STRA6 membrane receptor acts like a retinol channel. It is not yet clear whether it releases retinol to the membrane leaflet or loads it directly onto RBP1. (*d*) The uptake of carotenoids and other fat-soluble vitamins in mixed micelles into enterocytes of the gut is facilitated by scavenger receptors such as scavenger receptor class B type 1 (SR-B1). SR-B1 is also expressed on peripheral cells and facilitates the uptake of carotenoids and cholesterol from high-density lipoprotein (HDL). SR-B1 displays a hydrophobic tunnel that loads lipids into the membrane leaflet. (*e*) Nonvesicular transport of cholesterol and carotenoids between membranes is facilitated by GRAM-domain-containing proteins (GRAMD1 A, B, and C), also known as Aster proteins. Aster proteins display a tripartite structure with an N-terminal GRAM domain, a central StART-like lipid-binding domain, and a C-terminal membrane-anchoring domain.

Carotenoids and retinoids accumulate in photoreceptors of the retina at concentrations at least 1,000 times higher than those found in the bloodstream, and they exhibit distinct patterns of distribution within the retina. Recent advances have significantly enhanced our understanding of the factors governing the transport and distribution of these lipids in the body, as well as their preferential enrichment in ocular tissues. This research has unveiled key players, including transporters, binding proteins, and metabolic enzymes, responsible for carotenoid and retinoid homeostasis (Figure 2). Given that both excess and deficiency of carotenoids and retinoids can have detrimental effects on cells and tissues, the actions of these compounds must be tightly regulated and integrated into the broader context of cellular lipid metabolism. Not surprisingly, mutations in the genes encoding these essential components have been linked to a spectrum of vision-related disorders, ranging from night blindness to complex ophthalmic syndromes. In this review, we provide a summary and discussion of recent advancements in this field of research, with a particular emphasis on factors influencing the maintenance of ocular carotenoid and retinoid balance.

## 2. CAROTENOID AND RETINOID ABSORPTION AND PROCESSING IN THE INTESTINE

**Figure 3:**
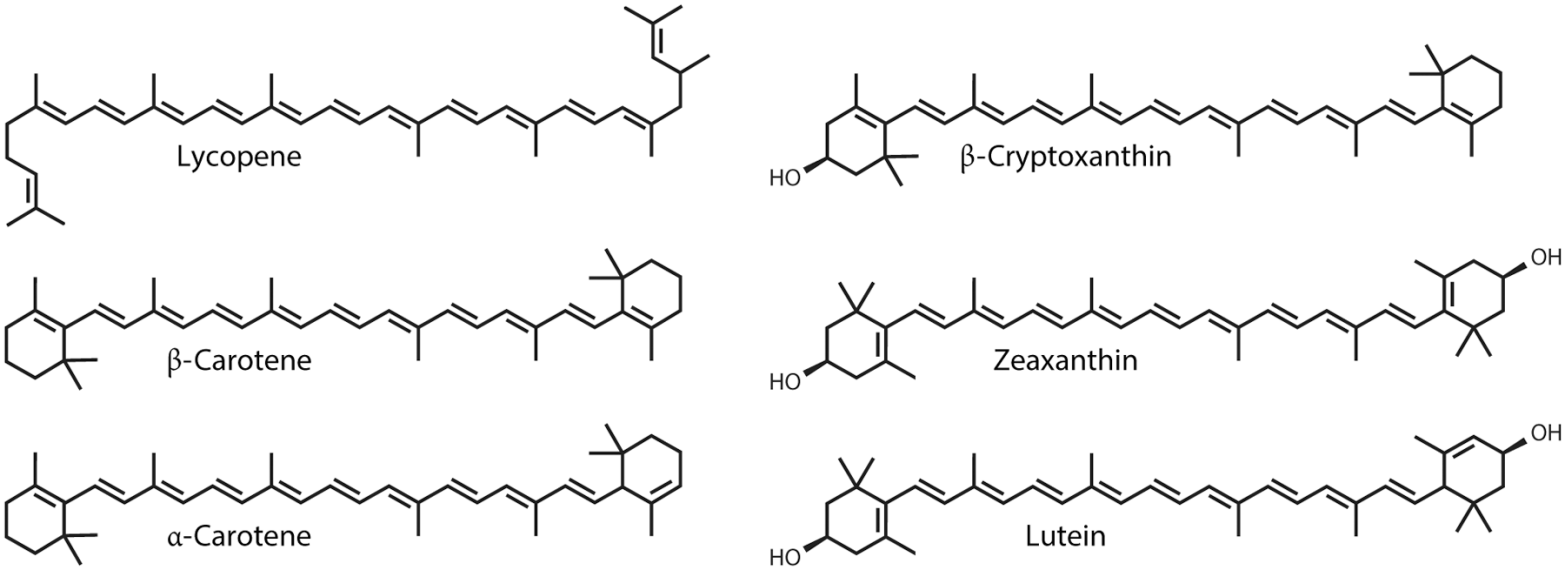
Structures of the most abundant carotenoids in the human blood.

The human diet typically includes approximately 50 carotenoids, with approximately 20 commonly found in human tissues. These carotenoids fall into two groups: carotenes (hydrocarbons) and xanthophylls (oxygenated carotene metabolites). Six primary carotenoids circulate in human blood: β-carotene, α-carotene, β-cryptoxanthin, lutein, zeaxanthin, and lycopene (Figure 3).

Dietary carotenoids are absorbed in the intestine, where they form mixed micelles with various compounds, including bile salts, cholesterol, fatty acids, monoacylglycerides, and phospholipids. The absorption of xanthophylls, often found as esters in fruits and vegetables, requires pancreatic carboxyl ester lipase to cleave the fatty acid moiety (Breithaupt et al. 2003). Carotenoid absorption is also influenced by factors such as the food matrix, formulation, and processing techniques (Bohn et al. 2017, Castenmiller & West 1998).

Dietary retinol is absorbed in the small intestine, while dietary REs require the action of enzymes like RE hydrolases (REHs) to convert them into free retinol (Borel & Desmarchelier 2017). The specific identity of the REH(s) involved in this reaction remains uncertain (D’Ambrosio et al. 2011).

Gastrointestinal and pancreatic diseases, such as cystic fibrosis, can affect carotenoid and retinoid absorption, potentially leading to visual impairment (Bonifant et al. 2014). Inflammatory conditions like Crohn’s disease and complications of bariatric surgery can also impact absorption (de Vries et al. 2018, Guerreiro & Ribeiro 2015). Further research, including studies in animal models, is crucial to understanding these effects and developing strategies to prevent micronutrient malabsorption, essential for eye health (de Vries et al. 2018).

Once dissolved in micelles, carotenoids undergo absorption by enterocytes, crossing the membrane bilayer for further metabolic processing within the brush border cells. Studies conducted using polarized CaCo-2 cells, which serve as a model for the human brush border membrane, have revealed that this process is both saturable and selective, implying the involvement of specific proteins (During et al. 2002).

The proteins responsible for carotenoid uptake have been identified as class B scavenger receptors. The significance of class B scavenger receptors in this regard was initially established in *Drosophila* (Kiefer et al. 2002). The discovery of the neither inactivation nor after potential D (ninaD) protein in a screen of *Drosophila* mutants lacking functional visual pigments offered the first evidence of the role of class B scavenger receptors in carotenoid and retinoid metabolism (Kiefer et al. 2002). Nonsense mutations in ninaD render flies deficient in carotenoids, retinoids, and tocopherols (Voolstra et al. 2006). In silkworms, a homologous scavenger receptor akin to ninaD has been identified as vital for acquiring dietary carotenoids, which are essential for silk coloration (Sakudoh et al. 2010).

In mammals, the scavenger receptor class B type 1 (SR-B1) was initially characterized in cholesterol uptake from HDLs (Acton et al. 1996). Later investigations highlighted SR-B1’s role in aiding the absorption of carotenoids from synthetic and mixed micelles (During et al. 2005, Reboul et al. 2005). Studies conducted in knockout mice have confirmed that SR-B1 plays a crucial role in carotene and xanthophyll absorption (van Bennekum et al. 2005, Widjaja-Adhi et al. 2015), as well as in absorption of other fat-soluble vitamins (Goncalves et al. 2014, Reboul et al. 2006). Additionally, cluster determinant 36 (CD36) contributes to the absorption of carotenoids from mixed micelles in the intestine (Borel et al. 2013, Shyam et al. 2017). Notably, canary birds rely on SR-B1 for carotenoid coloration of feathers and skin (Toomey et al. 2017). Mutant birds display white feather coloration and have markedly reduced carotenoid levels in their blood and tissues, leading to severe vitamin A deficiency (VAD).

In mammals, CD36 and SR-B1 are expressed predominantly in the proximal segments of the intestine, albeit at lower levels in distal regions (Lobo et al. 2001). The scavenger receptors are glycosylated transmembrane proteins featuring extensive extracellular domains. The crystal structure of lysosomal membrane protein 2, a member of the CD36 family, has been elucidated, revealing a large cavity spanning the entire protein length that serves as a conduit for lipid transfer from extracellular to cellular compartments (Rodrigueza et al. 1999, Yu et al. 2012). It was shown using plasmon resonance assays that SR-B1 and CD36 bind mixed micelles and that the binding depended on the micellar lipid composition (Goncalves et al. 2015). Carotenoids and other lipid substrates such as tocopherols and phylloquinones would then be liberated from the bound micelles and moved to the plasma membrane (PM) or the cytosol of the brush border cell via the internal channels of the scavenger receptors.

There has been a persistent interest in identifying membrane transporters that facilitate the uptake of retinol into enterocytes. While it is tempting to speculate that the absorption of retinol by enterocytes involves a membrane transporter, there is currently a lack of experimental evidence to support this notion. Recent research has, however, identified a membrane protein called retinol binding protein receptor 2 (RBPR2), which has the ability to bind to serum retinol binding proteins (RBPs) and assist in the entry of retinol into cells (Alapatt et al. 2013). The intestine expresses RBPR2, suggesting that this protein plays a potential role as a transporter of dietary retinol (Alapatt et al. 2013). Nonetheless, it remains uncertain whether RBPR2 is situated on the apical or basolateral membrane of enterocytes. If it is located on the basolateral membrane, then this would suggest that the receptor is involved in exchanging retinol with the bloodstream. Conversely, an apical localization of RBPR2 would indicate its capacity to recognize dietary retinol. Notably, recent efforts have led to the creation of *Rbpr2*-knockout mice (Radhakrishnan et al. 2022). Studies involving these mice may provide valuable insights into the role of RBPR2 in intestinal retinol metabolism.

### 2.1. The Processing of Carotenoids and Retinoids in Enterocytes

Carotenoids and retinoids must be distributed throughout the body for storage in the liver and uptake by the eyes. To facilitate this process, the absorbed precursors of vitamin A are metabolically transformed into REs and are integrated into chylomicrons alongside parent carotenoids and other dietary lipids (O’Byrne & Blaner 2013, O’Byrne et al. 2005). The assembly of chylomicrons takes place within the endoplasmic reticulum (ER) of enterocytes and relies on the presence of surface proteins, namely, apolipoprotein B48 (apoB48) and apolipoprotein A4. The microsomal triglyceride transport protein catalyzes the lipidation of apoB48 on the inner leaflet of the ER (Iqbal et al. 2008). These prechylomicrons are then transported to the Golgi apparatus, where they acquire apolipoprotein A1. Mature chylomicrons are subsequently secreted into the lymphatic system and enter the bloodstream at the subclavian vein.

Carotenoids and retinoids need to be transported in enterocytes and metabolically processed by enzymes. The hydrophobicity of the lipids imposes limitations on their solubility and diffusion within the aqueous compartments of cells. Therefore, the enterocytes express specific binding proteins for their transport and metabolic processing. Human RBP1–4 are members of the lipocalin family of lipid-binding proteins (Widjaja-Adhi & Golczak 2019). Among these, RBP2 stands out, as it exhibits high expression in the intestine, constituting up to 1% of cytosolic proteins within enterocytes (Blaner et al. 2020). It has been suggested that RBP2 may play a role in directing retinol to specific acyltransferases for esterification (Wongsiriroj et al. 2008). This esterification process occurs through the activity of either LRAT (lecithin:retinol acyl transferase) or DGAT (acyl CoA:diacylglycerol acyltransferase) (Wongsiriroj et al. 2014). DGAT1 utilizes coenzyme A as a supplier of acyl groups for RE production. LRAT, which belongs to the ancient NlpC/P60 thiol peptidase protein superfamily (Golczak et al. 2015, Pang et al. 2006), specifically relocates an acyl moiety from the sn-1 position of phosphatidylcholine. The structure of NlpC/P60 thiol peptidases bears a resemblance to papain-like proteases and comprises a four-strand antiparallel β-sheet and three α-helices. The conserved catalytic triad residues, Cys161, His60, and His72, define the active center. LRAT employs a similar catalytic mechanism to thiol peptidases, where the deprotonated Cys161 acts as a nucleophile, attacking the carbonyl carbon of an ester bond at the sn-1 position of phosphatidylcholine, eventually leading to the transfer of an acyl group and the trans-esterification of retinol (Golczak et al. 2015, Pang et al. 2012).

Studies involving mice with intestinal DGAT deficiency do not indicate impaired retinol esterification (Ables et al. 2012), suggesting that LRAT likely plays the primary role in the synthesis of intestinal REs. Additionally, LRAT plays a pivotal role in sustaining RA levels within the intestine, thereby regulating the retinoid signaling that is essential for enterocyte homeostasis (Ramkumar et al. 2021). Although RBP2 appears to be vital for retinol absorption, it is plausible that alternative trafficking pathways, involving alternative routes, may also exist. In fact, studies involving the gavage of wild-type and *Rbp2*-knockout mice with a physiological dose of retinol did not reveal a statistically significant difference in postprandial retinol and RE plasma concentrations or in the amount of retinoids accumulated in the duodenal mucosa of these mice at the time of sacrifice (Wongsiriroj et al. 2008).

The intracellular transportation of carotenoids can be achieved through two primary mechanisms: vesicular transport and nonvesicular transport facilitated by binding proteins. Vesicular transport entails the packaging of these lipids into various lipoprotein classes, including chylomicrons, very-low-density lipoproteins, low-density lipoproteins (LDLs), and HDLs, along with the receptor-mediated uptake of these lipoproteins’ cargo into cells in various tissues, as has recently been reviewed elsewhere (Miller et al. 2020, von Lintig et al. 2020). On the other hand, nonvesicular transport relies on specific binding proteins that facilitate the transfer of carotenoids between different cellular membranes. Carotenoid-binding proteins have been molecularly identified in organisms such as lobsters and silkworms (Cianci et al. 2002, Sakudoh et al. 2007, Slonimskiy et al. 2022). However, proteins for the transport of carotenoids within mammalian cells have not been identified. Recent research implicates Aster proteins in this process (Bandara et al. 2022). Aster proteins belong to the stARkin protein superfamily (Balla et al. 2019, Elbaz-Alon et al. 2015). Mammalian members of these sterol transfer proteins are known as GRAM-domain-containing (GRAMD1/2/3) proteins, and some of them localize at membrane contact sites between the ER and mitochondria, as well as contact sites between the ER and PM (Besprozvannaya et al. 2018). The GRAMD1/2/3 proteins feature an N-terminal GRAM domain that directly interacts with phospholipids in the PM. They also possess a C-terminal transmembrane helix anchored in the ER. Among GRAMD proteins, GRAMD1 gene products, namely, Aster A, Aster B, and Aster C, also contain a StART-like domain. Aster proteins were initially characterized in the context of cholesterol transport from the PM to the ER and to mitochondria (Sandhu et al. 2018). The crystal structure of murine Aster A with bound cholesterol has provided valuable insights into the lipid-binding properties of these proteins (Sandhu et al. 2018). Structural prediction revealed that the StART-like domain of Aster proteins is long enough to accommodate a rigid C40 carotenoid. Expression of the StART-like domain of Aster A and B proteins in a zeaxanthin-producing *Escherichia coli* strain uncovered that these proteins could extract the carotenoids from the bacterial membranes (Bandara et al. 2022, 2023). The carotenoprotein complexes displayed a yellow color and a spectral shift of the bound pigment. In vitro carotenoid binding assays demonstrated that carotenoids could compete with cholesterol for the lipid-binding cavity of Aster proteins (Bandara et al. 2022). Furthermore, these studies demonstrated that Aster A and Aster B showed a preference for binding lutein and zeaxanthin, with β-carotene exhibiting lower binding affinity (Bandara et al. 2023).

Studies in human A549 cells showed that Aster B was capable of transporting zeaxanthin to mitochondria. In contrast, the pure hydrocarbon β-carotene was not transported to the organelles, consistent with its metabolic conversion to vitamin A in the cytosol (Bandara et al. 2023). Notably, Aster B and Aster C are expressed in the enterocytes of the intestine. Recent research has linked Aster B to the transport of cholesterol in the enterocytes of the small intestine (Ferrari et al. 2023). Given the demonstrated carotenoid-binding capabilities of Aster proteins, it is reasonable to propose that these proteins may facilitate carotenoid transport between different cellular compartments in enterocytes. Investigations involving Aster B– and C–knockout mice will provide additional insights into this process and establish the role of this class of lipid-binding proteins in facilitating carotenoid transport.

**Figure 4:**
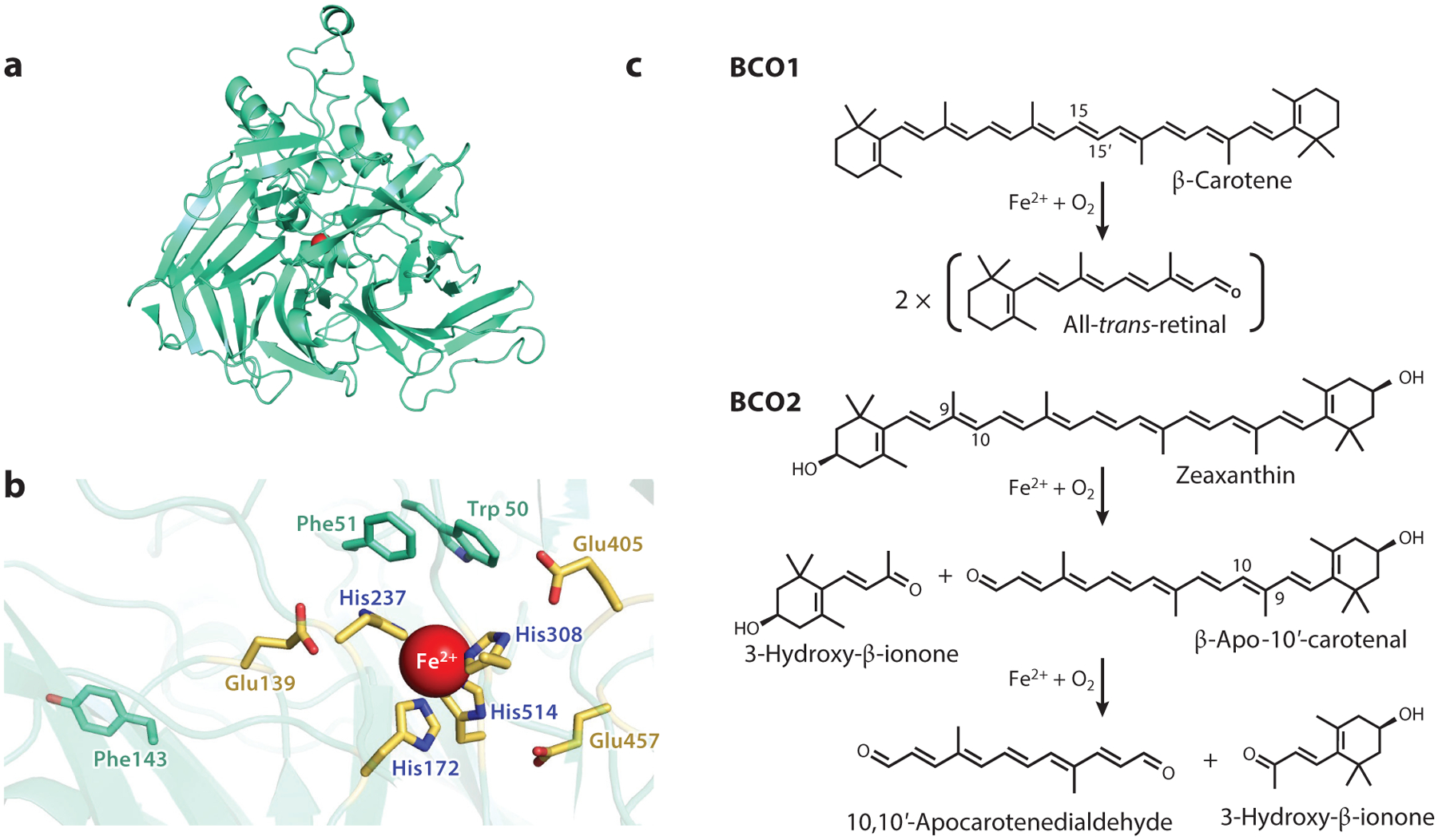
Carotenoid cleavage dioxygenases are critical for carotenoid homeostasis. (*a*) Structural model of human BCO1. The ferrous iron in the reaction center is highlighted in red. (*b*) Close-up image of the active site of BCO1. The active center contains a ferrous iron (highlighted in *red*) that is coordinated by four conserved histidine residues (*purple*) and glutamate residues (*yellow*). Additionally, aromatic amino side chains that interact with the carotenoid substrates are highlighted. (*c*) Representative reaction catalyzed by BCO1 and BCO2 with carotenoid substrates.

Two members of the carotenoid cleavage dioxygenase (CCD) protein family, known as β-carotene-oxygenase (BCO)1 and BCO2, are pivotal for carotenoid metabolism within enterocytes (Kelly et al. 2018) (Figure 4). BCO1, a cytosolic enzyme, is responsible for cleaving carotenoids and apocarotenoids (>C20) that possess at least one unsubstituted β-ionone ring at the C15-C15′ position. This cleavage yields two retinaldehyde molecules for provitamin carotenoid cleavage and a retinal and a short-chain apocarotenoid (<C20) for apocarotenoid cleavage (Amengual et al. 2013, Kelly et al. 2018). On the other hand, human and mouse BCO2 are mitochondrial enzymes that display a broad range of substrate specificity for various carotenoids and apocarotenoids, cleaving them across the C9′-C10′ double bond (Amengual et al. 2011, Bandara et al. 2021, Thomas et al. 2020). Unfortunately, three-dimensional crystal structures for BCO1 and BCO2 are currently unavailable, prompting the use of homology modeling with the aid of 3D structures of the retinal pigment RPE65 (Kiser et al. 2009) and a recently solved structure of a bacterial CCD containing a bound apocarotenoid substrate (Daruwalla et al. 2020).

In terms of structural characteristics, CCDs possess a seven-bladed β-propeller scaffold connected with α-helices and loops on top. These enzymes rely on ferrous iron as a cofactor, which is coordinated by four conserved histidine residues and three glutamate residues. The substrate-binding cavity of CCDs mainly consists of hydrophobic amino acids that position the scissile bond of carotenoids toward the ferrous iron in the active center. While BCO1 and BCO2 share a similar overall fold, their substrate specificities are distinct. Recent research has shown that BCO2 has a bipartite substrate-binding cavity that can accommodate a wide range of carotenoid and apocarotenoid substrates. The cleavage of a carotenoid by BCO2 proceeds in a stepwise manner through a long-chain apocarotenoid intermediate (Bandara et al. 2021) (Figure 4). In contrast, BCO1’s substrate tunnel has a narrow entrance that selects substrates with unsubstituted β-ionone rings, contributing to the specificity of vitamin A production (Kelly et al. 2018).

The development of mouse knockout models deficient in BCO1 and BCO2 has paved the way for a thorough examination of the contribution of BCO1 and BCO2 to carotenoid metabolism (Amengual et al. 2011, Hessel et al. 2007). These studies have highlighted the crucial role of BCO1 as a major enzyme for β-carotene metabolism and vitamin A production (Amengual et al. 2013). Similarly, humans with inherited BCO1 deficiency exhibit elevated β-carotene levels and reduced vitamin A levels (Lindqvist et al. 2007). The accumulation of β-carotene in *Bco1-*knockout mice can be attributed to the biochemical properties of Aster proteins, which favor xanthophyll over carotene in transport to the mitochondria (Bandara et al. 2023), where BCO2 is located (Palczewski et al. 2014). Consequently, carotenes are retained in the cytosol in lipid droplets in BCO1 deficiency and do not enter the mitochondria for processing by BCO2 (Palczewski et al. 2014, 2016). Therefore, BCO1-deficient mice accumulate β-carotene and only display trace amounts of the BCO2 cleavage product β-10′-apocarotenol (Amengual et al. 2013).

*Bco2*-knockout mice accumulate lycopene, β-cryptoxanthin, zeaxanthin, lutein, and astaxanthin (Amengual et al. 2011, 2013; Bradley et al. 2023; Kelly et al. 2018). In wild-type mice, high expression of BCO2 in enterocytes prevents absorption of xanthophyll and body distribution to peripheral tissues (Thomas et al. 2023). In contrast, many mammals, including humans, absorb carotenoids intact and accumulate them in specific tissues, including the eyes. This accumulation might be explained by the presence or absence of BCO2 expression in intestinal enterocytes. While BCO2 is highly expressed in the mouse intestine, human enterocytes do not express the enzyme in significant amounts (Thomas et al. 2023).

The significance of BCO2 in carotenoid homeostasis has been demonstrated in various animal species. Genetic variants and polymorphisms in BCO2 have been reported in rabbits, sheep, and bovine species. Mutations in the bovine and sheep BCO2 genes lead to distinct phenotypes affecting fat and milk color (Berry et al. 2009), while genetic polymorphisms in BCO2 are responsible for color variations in domestic chickens (Eriksson et al. 2008).

### 2.2. Control of Carotenoid Absorption and Vitamin A Production in Intestinal Enterocytes

**Figure 5:**
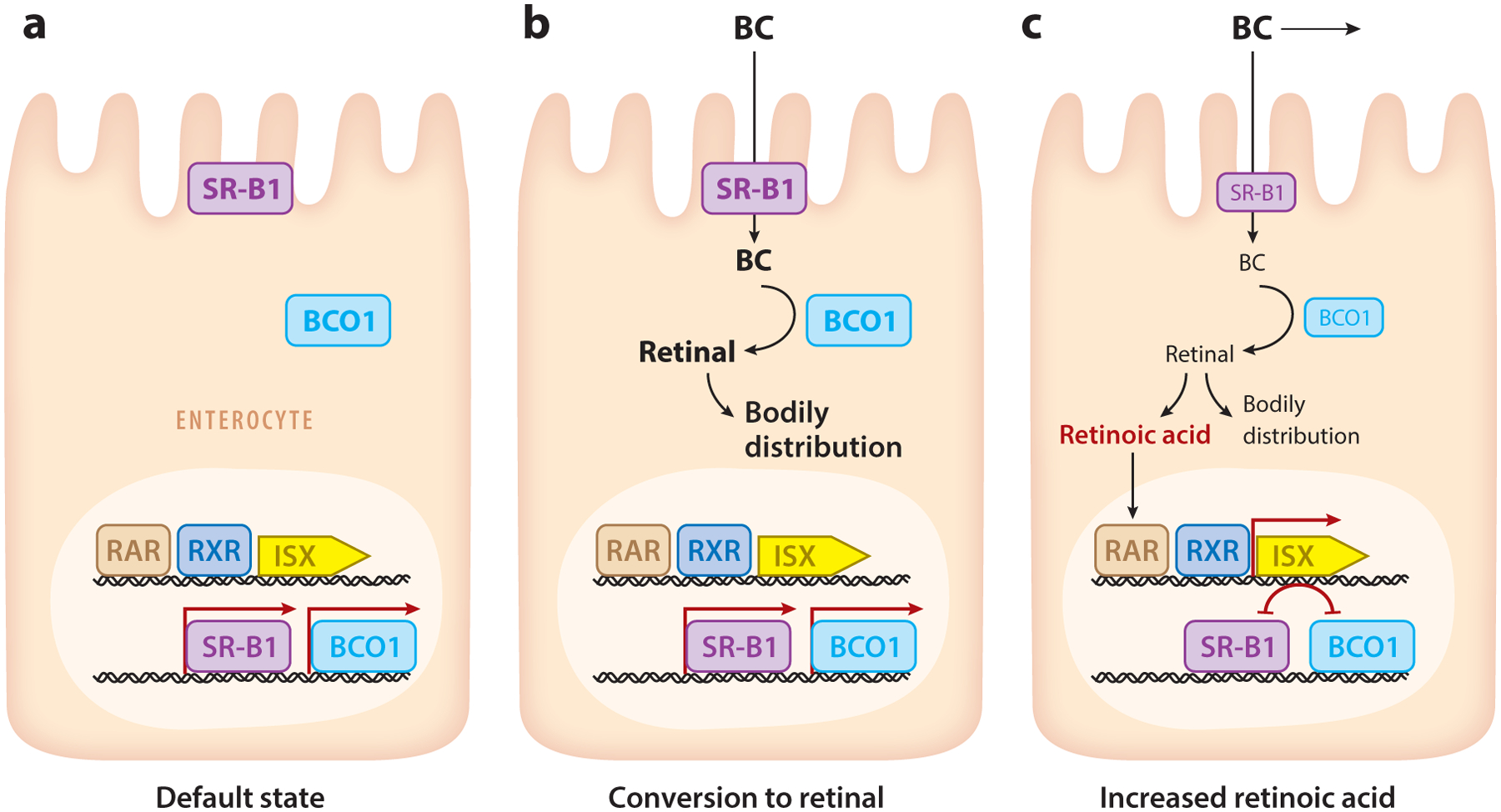
Regulation of β-carotene (BC) absorption and conversion to vitamin A. (*a*) In the default state, the enterocytes express high levels of scavenger receptor class B type 1 (SR-B1) and BC-oxygenase-1 (BCO1). (*b*) When BC becomes available in the diet, the nutrient is readily absorbed and converted to retinal. Retinal is reduced, esterified, and transported as retinyl ester in chylomicrons for bodily distribution. (*c*) The levels of retinoic acid eventually rise in enterocytes through sustained retinal production. Retinoic acid binds to retinoic acid receptors (RARs) that, in conjunction with RXR receptors, induce the expression of the homeodomain transcription factor ISX. In turn, ISX suppresses messenger RNA expression of the genes encoding SR-B1 and BCO1, respectively.

Numerous studies have presented compelling evidence regarding the influence of an individual’s vitamin A status on carotenoid absorption and the conversion of carotenoids into vitamin A. It is now widely accepted that vitamin production is subject to negative feedback control (Figure 5). Within enterocytes, the *Bco1* gene is highly expressed in the vitamin A–deficient state. However, in the presence of preformed vitamin A or other vitamin A precursors in the diet, enterocytes quickly cease *Bco1* expression and vitamin A production (Bachmann et al. 2002). Similarly, when the body’s vitamin A stores are replete, the expression of the *Bco1* gene in brush border cells is repressed. This regulatory mechanism prevents excessive vitamin A production, which can be harmful to the organism. Therefore, REs, but not provitamin A, can be toxic when present in vast amounts in the diet, i.e., in the livers of polar bears and seals (Senoo et al. 2012).

The repressor protein responsible for controlling vitamin A production from carotenoids has been identified as the intestine-specific homeobox transcription factor ISX (Seino et al. 2008). The repressor’s activity is regulated by RA (Lobo et al. 2010, 2013; Ramkumar et al. 2021). The acidic form of vitamin A binds to RA receptors, leading to the induction of *Isx* messenger RNA (mRNA) expression in the intestine. In turn, ISX binds to the promoters of the *Bco1* and *SR-B1* genes, repressing their mRNA expression (Lobo et al. 2013, Widjaja-Adhi et al. 2015). RA concentrations in enterocytes are controlled by the LRAT enzyme, which directs the metabolic flow of retinoids toward RE formation and integration into chylomicrons (Ramkumar et al. 2021). This role has been demonstrated in *Lrat*-knockout mice, which cannot balance intestinal RA levels and become severely vitamin A deficient when provided with β-carotene as their sole dietary source of retinoids. Accordingly, the phenotype of LRAT-deficient mice can be rescued by antagonists of RARs that restore BCO1 expression or by knocking out the gene encoding ISX (Ramkumar et al. 2021).

Data from knockout mice support the notion of controlled vitamin A production (Lobo et al. 2013, Seino et al. 2008, Widjaja-Adhi et al. 2017); however, the fine-tuning of this regulation and its applicability to other mammals remain to be established in more detail (Sowa et al. 2020). In humans, significant single-nucleotide polymorphisms (SNPs) have been identified in the genes encoding BCO1, SR-B1, and the transcription factor ISX (Borel & Desmarchelier 2018). These polymorphisms affect circulating carotenoid levels (Ferrucci et al. 2009). Interestingly, SNPs in the *BCO1* gene are also associated with macular pigment density (Meyers et al. 2013). This observation may seem surprising, since xanthophylls are not substrates for the BCO1 enzyme. However, the ISX-dependent regulation of SR-B1 expression in the intestine explains the interaction between xanthophyll and β-carotene (Thomas et al. 2023, Widjaja-Adhi et al. 2015). This concept has been demonstrated in mice deficient for ISX and BCO2. These compound mutants accumulate significantly higher concentrations of zeaxanthin in peripheral tissues than mice deficient for BCO2 alone. This enhanced accumulation appears as a golden skin phenotype in an ISX- and BCO2-deficient albino mouse (Thomas et al. 2023).

## 3. CAROTENOID TRANSPORT TO THE EYES

The levels of circulating carotenoids are subject to a multitude of factors, including individual genetics, health status, dietary intake, and vitamin A status (Bohn et al. 2017). Typically, these levels also exhibit correlations with serum triglyceride and cholesterol levels. Additionally, several SNPs in proteins influencing lipid and lipoprotein metabolism have been associated with carotenoid blood levels, indicating significant interactions between carotenoid and lipid metabolism (Borel & Desmarchelier 2018, Miller et al. 2020). Interactions between carotenoid and lipid metabolism have also been confirmed in mice (Palczewski et al. 2016). Another significant determinant of circulating carotenoid levels is body composition. Various studies have revealed a negative relationship between body mass index and plasma carotenoid concentrations (Bohn et al. 2017). A closer examination of this connection indicates that plasma carotenoid concentrations are inversely associated not only with fat mass, but also with lean body mass, suggesting that nonfat tissues, such as muscle, can also act as reservoirs for carotenoids. Notably, individuals with anorexia nervosa tend to exhibit exceptionally high plasma carotenoid levels, likely due to heightened mobilization in this catabolic condition (Curran-Celentano et al. 1985).

Within the bloodstream, carotenoids are not uniformly distributed among lipoprotein classes. Zeaxanthin and lutein, for example, exist in higher concentrations within HDLs compared to LDLs and very-low-density lipoproteins (Palczewski et al. 2016, Thomas & Harrison 2016). The content and composition of carotenoids also vary between different tissues. The high concentration of macular pigments in the retina, as compared to blood levels, implies the existence of specific transport mechanisms for carotenoids. Research involving human retinal pigment epithelial cell lines has indicated that the scavenger receptor SR-B1 plays a role in carotenoid uptake from HDLs (Thomas & Harrison 2016). SR-B1 is expressed in several tissues, including the liver, intestine, macrophages, adrenal glands, testes, and ovaries. The involvement of HDLs and SR-B1 in ocular carotenoid uptake has been recently demonstrated in mice. Mice deficient in BCO2 and apolipoprotein A1, the major lipoprotein of HDLs, showed reduced zeaxanthin and lutein levels in ocular tissues (Li et al. 2022). Thus, emerging evidence indicates that SR-B1 is involved in the accumulation of macular pigments at the outer blood–brain barrier in the RPE.

β-Carotene present in circulating LDLs is absorbed through LDL receptor–mediated endocytosis into the RPE (Thomas & Harrison 2016). Human retinal pigment epithelial cells express BCO1, the enzyme responsible for vitamin A formation, suggesting that local vitamin A synthesis may contribute to ocular retinoid homeostasis (Chichili et al. 2005).

Once taken up by the RPE, macular pigments must be transported to the retina and amassed in the fovea lutea. The macular pigment concentration is approximately 100-fold higher in the fovea region than in the peripheral retina. The distributions of zeaxanthin and lutein were determined by an elegant confocal Raman spectroscopy approach (Li et al. 2020). Zeaxanthin concentration is high in the fovea centralis and enriched in the Henle’s fiber layer of the fovea. This structure contains bundles of unmyelinated cone photoreceptor axons that connect to ganglion cells in the outer plexiform layer of the retina. In contrast, lutein distributes more diffusely in the central retina and at a lower concentration.

It has been proposed that carotenoid-binding proteins play a role in the retention of macular pigments (Bernstein et al. 2016). Among these, glutathione S-transferase pi isoform (GSTP1) has been suggested as a zeaxanthin-binding protein (Bhosale et al. 2004), and StAR-related lipid transfer domain-containing 3 (StARD3) is believed to function as a lutein-binding protein (Li et al. 2011). StARD3 bears a resemblance to carotenoid-binding proteins found in silkworms. However, our understanding of the precise mechanism of carotenoid binding at the atomic level remains incomplete. GSTP1 displays a globular enzyme structure that lacks a dedicated lipid-binding cavity for carotenoid binding. On the other hand, StARD3, known for its role in transferring cholesterol between endosomes and mitochondria, has a lipid-binding cavity that is relatively short and unable to bind C40 carotenoid molecules (Sluchanko et al. 2022).

Additionally, RBP3, also known as interphotoreceptor retinoid-binding protein, has been proposed to bind carotenoids (Vachali et al. 2013). RBP3 is recognized as a retinoid transporter, facilitating the movement of the chromophore between RPE and photoreceptor cells (Saari et al. 1985).

In contrast, the StART-like domain of Aster B effectively binds zeaxanthin and lutein and transports them to the mitochondria of cells (Bandara et al. 2023). The expression of the *GRAMD1B* gene, encoding Aster B, is notably high in the human retina, as indicated by RNA and protein analysis of donor eye samples (Bandara et al. 2022). This expression is absent in mouse retinas, which do not accumulate carotenoids. Immunohistochemistry confirmed the presence of Aster B in both central and peripheral regions of the human retina. Staining for Aster B is detected in rod and cone photoreceptors, as well as other cell types within the retina (Bandara et al. 2022). The pattern of Aster B immunostaining in the retina, coupled with its proposed role as a carotenoid transport protein, raises the question of why carotenoids tend to accumulate in central areas of the retina. A possible answer emerged from single-cell sequencing that analyzed the transcriptomes of cone photoreceptors from peripheral and central regions of the retina. This study identified BCO2 as one of the most regulated genes (Voigt et al. 2019). Therefore, one may speculate that GRAMD1B transports and augments macular pigments in retinal cells, leading to their increased concentration in the retina. In the peripheral regions of the retina, mitochondrial BCO2 cleaves the macular pigments into apocarotenoids and disposes of them. However, in the central region, reduced BCO2 expression permits the accumulation of the pigments. Thus, high levels of Aster B expression and the differential expression of BCO2 offer a mechanism for establishing the distribution of carotenoids within the human retina (Bandara & von Lintig 2022).

Remarkably, photoreceptors contain clusters of mitochondria, even though they primarily rely on aerobic glycolysis for energy production (Hurley 2021). Aster B facilitates macular pigment accumulation in mitochondria, suggesting that the organelles play a special role as carotenoid storage compartments in the central regions of the human retina. Supporting this notion, histological analysis of the fovea in patients with macular telangiectasia type 2 revealed damaged mitochondria with disrupted inner membranes (Zucker et al. 2020). Notably, mitochondrial inner membranes are the primary sites for carotenoid accumulation (Palczewski et al. 2014), and the disease is characterized by a dramatic reduction in macular pigment density (Charbel Issa et al. 2009).

## 4. VITAMIN A TRANSPORT IN THE BLOOD AND UPTAKE IN THE EYES

**Figure 6:**
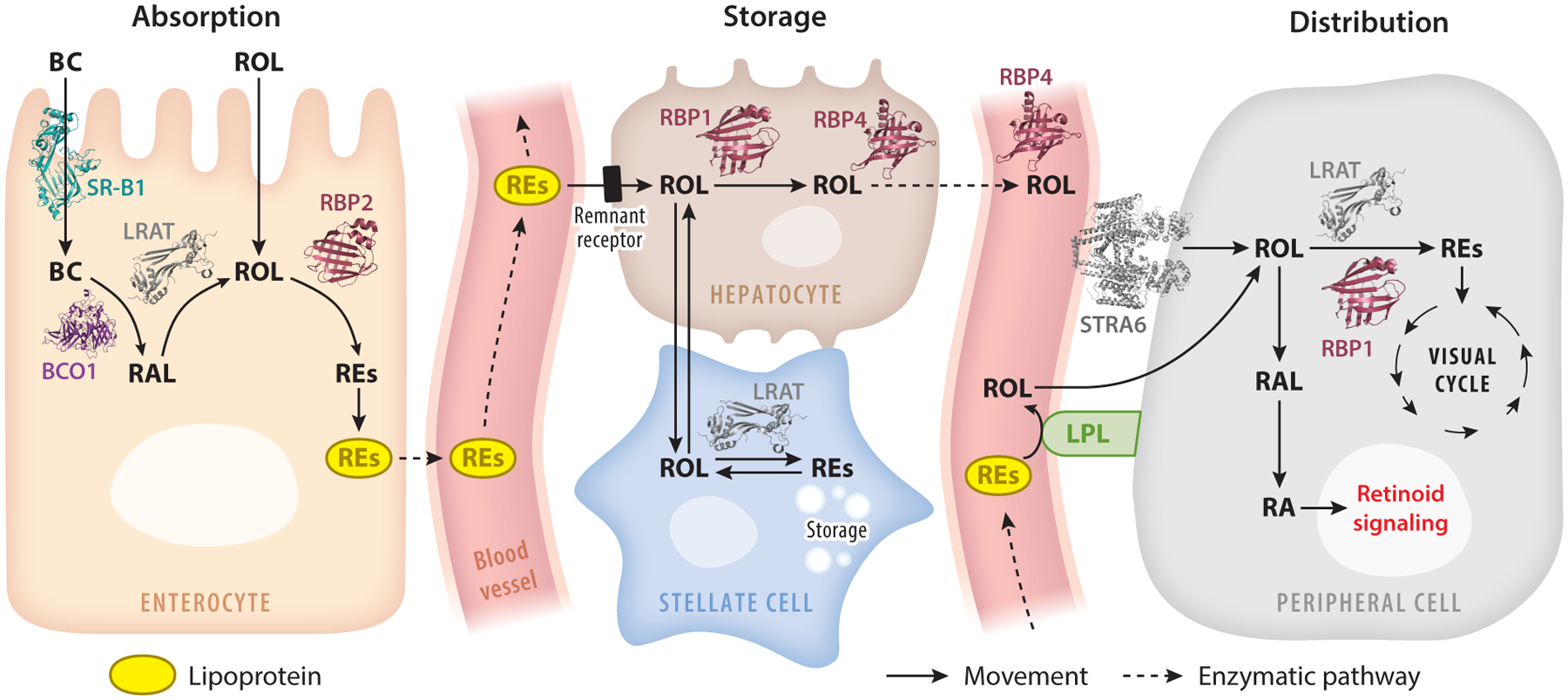
Scheme of the biosynthesis of retinoids and their transport and storage in the mammalian body. Major enzymes, transport proteins, and metabolites of the process are indicated in the figure. Abbreviations: BC, β-carotene; BCO1, β-carotene-oxygenase 1; LPL, lipoprotein lipase; LRAT, lecithin:retinol acyltransferase; RA, retinoic acid; RAL, retinal; RBP1,2,4, retinol binding protein 1, 2, and 4; REs, retinyl esters; ROL, retinol.

Chylomicrons transport dietary lipids, including REs (Figure 6). Upon secretion into the bloodstream the lipoproteins interact with peripheral tissue lipoprotein lipase, leading to the liberation of lipids and the uptake of carotenoids and retinol into peripheral tissues (He et al. 2018). Studies in mice showed that this pathway proves effective in maintaining retinoid homeostasis in many tissues, including the developing embryo and fetus, when they are nourished with retinoid-rich diets (Amengual et al. 2014, Berry et al. 2013, Quadro et al. 1999) (Figure 6).

Upon interaction with lipoprotein lipase, chylomicron remnants are formed and subsequently taken up by the liver via remnant receptors. In hepatocytes, REs are hydrolyzed by a yet unidentified REH into fatty acids and retinol. Retinol is transported to stellate cells, a process that involves cellular RBP1, encoded by the *RBP1* gene. Evidence supporting this comes from the phenotype of *Rbp1*-knockout mice, which exhibit reduced hepatic RE stores (Ghyselinck et al. 1999). In stellate cells, retinol is re-esterified by LRAT and stored in lipid droplets (O’Byrne et al. 2005).

The hepatic vitamin A stores play a crucial role in maintaining stable vitamin A concentrations in the bloodstream. All-*trans*-retinol bound to RBP4, also known as RBP, serves as the primary transport vehicle (Figure 6). RBP4 is primarily produced in hepatocytes (Thompson et al. 2017), and its release into the circulation depends on the presence of vitamin A (Quadro et al. 1999). In the bloodstream, holo-RBP4 forms a ternary complex with transthyretin, preventing the glomerular filtration of the relatively small holo-RBP4 protein in the kidney (Episkopou et al. 1993).

For a long time, the way in which retinol, in its holo-RBP4 transport form, crosses cell membranes and enters target cells remained a subject of controversy. Biochemical studies hinted at the existence of a specific receptor for the uptake of vitamin A from holo-RBP4 (Heller & Bok 1976). Three decades after the initial biochemical description of the RBP4 receptor, it was identified as the *STRA6* gene product (Kawaguchi et al. 2007), a membrane protein previously recognized as an RA-inducible protein (Bouillet et al. 1997). Experiments in cell lines demonstrated that STRA6 fulfilled all criteria of a bona fide RBP4 membrane receptor: (*a*) high-affinity binding of RBP4; (*b*) mediation of cellular uptake of vitamin A; and (*c*) expression in tissues, such as the RPE, choroid plexus of the brain, and Sertoli cells of the testis (Kawaguchi et al. 2007). Shortly after its discovery, a zebrafish study showed that knockdown of STRA6 led to reduced retinoid concentrations in the larval eyes, providing physiological evidence for STRA6’s role in vitamin A transport (Isken et al. 2008).

Structural analysis revealed that zebrafish STRA6 assembles as a complex dimer with 18 transmembrane helices (nine per protomer) and two long horizontal intramembrane helices that interact at the dimer core (Chen et al. 2016). The receptor complex features a lipophilic cleft to which holo-RBP4 binds with high affinity (Chen et al. 2016, Kawaguchi et al. 2007).

Studies conducted in cell lines demonstrate that STRA6 facilitates the bidirectional flux of retinol between RBP4 and cells (Isken et al. 2008, Kawaguchi et al. 2011). The opening and closing of this channel are regulated processes and involve calcium signaling and calmodulin, which is bound to the STRA6 dimeric complex (Chen et al. 2016, Zhong et al. 2020). The binding is regulated by the physiological calcium concentration; however, the details of this regulation are yet not fully understood (Young et al. 2021).

LRAT works downstream of STRA6 in vitamin A uptake (Amengual et al. 2012). Thus, the esterification serves as a trap for retinol that amasses the lipid in cells such as the RPE. Consequently, genetic disruption of the *Lrat* gene leads to blindness in mice due to their inability to acquire vitamin A from circulating RBP4 (Amengual et al. 2012) and to produce REs for visual chromophore production through the canonical retinoid cycle (Batten et al. 2004).

At young ages, *Stra6*-knockout mice exhibit very low ocular vitamin A levels and display significantly reduced electroretinogram (ERG) responses (Amengual et al. 2014, Berry et al. 2013, Ruiz et al. 2012). Blood and other tissues, such as the lungs, fat, and liver, maintain normal vitamin A levels through chylomicrons in mice raised on vitamin A–rich chow (Amengual et al. 2014, Berry et al. 2013). Young *Stra6*-knockout mice display an imbalance between rod opsins and chromophores, resulting in large amounts of unliganded opsins in their photoreceptors (Ramkumar et al. 2022b). Ocular vitamin A levels increase with age when mice are nourished with vitamin A–rich chow (Amengual et al. 2014). This increase restores rod photoreceptor ERG responses and improves the ratio between rod opsins and chromophores in photoreceptors (Ramkumar et al. 2022b). Nonetheless, the levels of ocular retinoids of STRA6-deficient mice remain consistently reduced throughout their entire life cycle (Ramkumar et al. 2022b). A parallel phenomenon has been observed in RBP4-deficient mice (Montenegro et al. 2022, Quadro et al. 1999).

Remarkably, there are noticeable alterations in the expression of photoreceptor marker genes in STRA6 deficiency (Ramkumar et al. 2022b). Specifically, marker genes associated with cone photoreceptors are affected. Despite an increase in ocular retinoid concentrations with age, cone photoreceptors in these mice consistently exhibit absent or mislocalized cone opsins (Ramkumar et al. 2022b). Given that rods outnumber cones in the retina, this scenario seemingly creates competition for the limited chromophore supply that is particularly unfavorable for cones. These observations underscore the fact that lipoprotein-dependent delivery pathways for vitamin A cannot effectively substitute for the RBP4–STRA6 system in the eyes.

Treatment with pharmacological doses of vitamin A improves the rod phenotype but does not restore cone photoreceptor responses (Ramkumar et al. 2022b). However, intervention with β-carotene in STRA6-deficient mice enhances the supply of chromophore to cone photoreceptors (Moon et al. 2022b). Relieving the intrinsic negative feedback regulation of β-carotene conversion in the intestine through *Isx* knockout further enhances cone photoreceptor function in STRA6-deficient eyes (Moon et al. 2022b). The favorable effects of β-carotene on cones likely stem from improved serum kinetics of REs in lipoproteins that mitigate the competition for chromophores between rod and cone photoreceptors. However, the improvement comes at the expense of excess retinoid accumulations in other tissues (Moon et al. 2022b). Together, these studies emphasize the critical role of STRA6 in regulating ocular vitamin A homeostasis and delivery of chromophores to photoreceptors.

Studies in mice revealed that STRA6 and RBP4 also provide unique advantages for enduring extended periods of VAD (Kelly et al. 2016). The eyes, in particular, derive significant benefits from this system. Mouse eyes, even after 20 weeks of VAD, maintain normal vitamin A levels, while vitamin A stores in the liver and lungs are nearly depleted under these conditions (Amengual et al. 2012). Only after eight months of dietary deprivation do significant losses of ocular vitamin A occur in mice (Hu et al. 2011). In the absence of STRA6, however, the eyes are susceptible to VAD (Kelly et al. 2016).

To gain deeper insights into the consequences of ocular VAD, a study subjected both wild-type and STRA6-deficient mice to diets with and without vitamin A supplements (Moon et al. 2023). After a 12-week-long dietary intervention, RNA was isolated from eyecups, and RNA sequencing analysis was conducted. This comparative analysis revealed the transcriptomes of STRA6-deficient and wild-type eyes under different supply conditions for the nutrient (Moon et al. 2023).

In mild VAD, the differentially expressed genes were associated with components of the cone phototransduction machinery. Additionally, mRNA levels of genes encoding components involved in oxidative stress responses were changed (Moon et al. 2023). Under conditions of severe VAD, the top enriched biological processes included epithelium development, biological adhesion, and cell–cell junction organization. Among the differentially expressed genes were *plakophilin-1* and *desmocollins*, which encode cell junction proteins. A disruption of the barrier function of the RPE in STRA6-deficient mice was confirmed by zonula ocludens-1 staining and tests for barrier leakage (Moon et al. 2023). Anomalies were primarily detected in STRA6-deficient RPE tissues under VAD conditions. This finding suggests that severe ocular VAD significantly affects the structural integrity of the outer blood–retinal barrier (BRB). Previously, a role of retinoid signaling for the maintenance of the outer BRB was established in zebrafish larvae (Pollock et al. 2018). The study in STRA6-deficient mice broadened our understanding of and implicated STRA6 in this process (Moon et al. 2023). Interestingly, STRA6 deficiency also worsens ocular pathologies of diabetic mice (Ramkumar et al. 2022a), which are known to display characteristic changes in the BRB (Daruich et al. 2018).

The preferential transportation of stored vitamin A to the eyes is a result of the regulation of *STRA6* gene expression, which varies depending on the tissue type and the vitamin A status (Kelly et al. 2016). STRA6 is prominently expressed in epithelial tissues forming blood–tissue barriers, particularly in the RPE, Sertoli cells of the testis, and ependymal cells of the choroid plexus (Amengual et al. 2014, Berry et al. 2013, Kelly et al. 2016). In the eyes, where the demand for vitamin A is highest in the body, the expression of the *Stra6* gene remains unaltered by the retinoid status (Kelly et al. 2016). This explains why the maintenance of ocular retinoid balance can persist over extended periods of dietary VAD (Kelly et al. 2016). In the testis and brain, *Stra6* expression surpasses that in most other tissues, but it is positively regulated by retinoids (Kelly et al. 2016). This regulation seems to boost *Stra6* expression when there is an abundance of vitamin A, yet it reduces the consumption of vitamin A in these tissues when the dietary supply is restricted, thereby preserving stored retinoids for use in the eyes (Kelly et al. 2016).

On the other hand, peripheral tissues like the lungs and adipose tissues exhibit minimal STRA6 expression and seem to rely on the receipt of postmeal dietary vitamin A through lipoproteins, which they can store (Shmarakov et al. 2023). Nevertheless, some tissues may also serve as rapid removers of excess vitamin A from the bloodstream, since *Stra6* gene expression is responsive to retinoid signaling (Bouillet et al. 1997). This regulatory mechanism was demonstrated in ISX-deficient mice fed a diet rich in β-carotene (Moon et al. 2022a). In this supplementation scenario, the mice generated substantial quantities of retinoids due to uncontrolled expression of BCO1 and SR-B1 in enterocytes. This led to a swift buildup of retinoid reserves in the liver and peripheral tissues. The heightened storage of retinoids in peripheral tissues like the lungs is accompanied by an increase in STRA6 and LRAT expression driven by retinoid signaling (Moon et al. 2022a). Thus, the STRA6-mediated cellular uptake of vitamin A, combined with LRAT activity, adds an extra layer of control and precision in tissues with high vitamin A demands (Amengual et al. 2012, Moon et al. 2022a). These discoveries suggest that STRA6 functions akin to a cellular faucet, enabling enhanced cellular absorption of circulating vitamin A compared to passive diffusion (Sun & Kawaguchi 2011). The intricate regulation of STRA6 expression in peripheral tissues ensures the proper distribution of vitamin A, which is determined by the specific requirements of each tissue and the availability of nutrients in conditions of VAD or an excess of retinoid supply.

In humans, 24 missense and nonsense mutations in the *STRA6* gene have been identified as causing a severe microphthalmic syndrome known as Matthew-Wood syndrome (MWS) (Blaner 2007, Golzio et al. 2007). MWS is characterized by severe bilateral microphthalmia, often accompanied by pulmonary dysplasia, cardiac defects, diaphragmatic hernia, and other anomalies and malformations. The symptoms of MWS align with the central role of retinoids in mammalian embryonic development. However, the severity of these symptoms can vary, even within the same family, ranging from isolated microphthalmia to more severe syndromes (Casey et al. 2011). Similarly, mutations in the *RBP4* gene can lead to congenital eye malformations, including microphthalmia (Chou et al. 2015). Consistent evidence indicates that variations in the extraocular phenotype associated with *STRA6* mutations may be influenced by maternal vitamin A status and its delivery to the fetus. Studies in mice indicate that both dietary and stored vitamin A can be transported through the fetal–maternal blood barrier. Notably, dietary vitamin A restriction in *Rbp4*-knockout mice results in malformations that resemble the birth defects seen in MWS patients (Kim et al. 2008).

Biochemical evidence suggests that an RBP4 receptor is expressed in the placenta (Redondo et al. 2008). The identity of the placental receptor in mice remains uncertain, with conflicting evidence suggesting it could be either STRA6 or the recently discovered RBPR2. Both RBP4 receptors are present in this tissue, as has been documented in several studies (Alapatt et al. 2013, Kawaguchi et al. 2007, Kim et al. 2008). RBPR2 structurally resembles STRA6 and exhibits a binding site for RBP4. Functional studies with holo-RBP4 have demonstrated that this receptor facilitates the accumulation of cellular retinol (Alapatt et al. 2013, Solanki et al. 2020). Notably, the expression patterns of RBPR2 differ from those of STRA6 in several tissues, implying that these two proteins may play distinct roles in maintaining vitamin A homeostasis (Alapatt et al. 2013).

The presence of RBPR2 in the liver suggests its involvement in the reuptake of vitamin A bound to circulating holo-RBP4. Intriguingly, when ISX- and STRA6-deficient mice, as well as double mutant mice, were fed with β-carotene, hepatic RBPR2 expression increased (Moon et al. 2022a). This condition led to higher hepatic retinoid levels, suggesting that elevated hepatic RBPR2 plays a crucial role in controlling retinoid homeostasis under these circumstances.

A recent development is the creation of an RBPR2-deficient mouse model (Radhakrishnan et al. 2022). A study reports that these mice exhibit an ocular phenotype similar to STRA6-deficient mice, characterized by reduced ocular retinoid levels and impaired visual function. This impairment becomes more pronounced when the mice are subjected to vitamin A deprivation. It is worth noting that RBPR2 is not naturally expressed in mouse eyes, adding to the intrigue. Consequently, the specific biochemical mechanisms underlying the RBPR2-deficient phenotype require further investigation in the future. The generation of compound knockout mice that lack both RBP4 receptors will offer valuable insights into the interplay between these receptors in regulating vitamin A homeostasis in the eyes and other tissues.

## SUMMARY POINTS

1. Carotenoid absorption and body distribution are influenced by host-related factors, including individual genetics and vitamin A status.
2. Specific membrane receptors for carotenoid and retinoid uptake have been identified, and animal models can help researchers study their roles in the etiology of ophthalmic disease states.
3. Specific binding proteins facilitate nonvesicular transport of carotenoids and retinoids between membrane compartments.
4. The carotenoid cleavage dioxygenases β-carotene-oxygenase (BCO)1 and BCO2 play a crucial role in converting carotenoids to apocarotenoids, including retinoids, affecting the pigments’ cellular and tissue homeostasis.
5. A diet-responsive regulatory network, involving the transcription factor ISX, controls the conversion of β-carotene to retinaldehyde by the enzyme BCO1 in the intestine. Retinoic acid levels and retinoid signaling in enterocytes affect the balance of other carotenoids by controlling the levels of scavenger receptor class B type 1 (SR-B1).
6. Mitochondria are a significant site for carotenoid processing and accumulation within cells.
7. SR-B1, the GRAMD1B gene product, and BCO2 have been identified as major biochemical components of macular pigment metabolism. Their ocular expression patterns correlate with the accumulation of macular pigments in the human retina.
8. Stimulated by retinoic acid 6 (STRA6) serves as the receptor for holo-retinoid binding protein (RBP)4 and plays a critical role in maintaining ocular vitamin A uptake homeostasis under various supply conditions for the nutrient.

## FUTURE ISSUES

1. The mechanism by which the ISX-dependent control of vitamin A production is fine-tuned needs to be established. Is this response mainly critical to avoid excess retinoid production, or does it also regulate homeostasis under low and moderate supply conditions?
2. Is there any crosstalk between the liver and gut in the control of bodily retinoid stores?
3. Which signals control the opening and closing of the retinoid channel STRA6, and does this channel contribute to the dynamic influx and efflux of ocular retinoids?
4. Do mitochondria in cone photoreceptors provide a storage compartment for macular pigments?
5. What is the metabolic fate of apocarotenoids produced by BCO2 in the eyes and other tissues?
6. Do mutations and genetic polymorphism in *GRAMD* genes affect carotenoid metabolism in humans?
7. What is the precise role of RBP receptor 2 in retinoid homeostasis?
